# Methamphetamine exposure and chronic illness in police officers: significant improvement with sauna-based detoxification therapy

**DOI:** 10.1177/0748233711425070

**Published:** 2012-09

**Authors:** Gerald H Ross, Marie C Sternquist

**Affiliations:** 1 Utah Meth Cops Project, c/o American Detoxification Foundation, Salt Lake City, UT, USA; 2 Foundation for Advancements in Science and Education, Pasadena, CA, USA

**Keywords:** Methamphetamine, police, chemical exposure, contamination, sauna, detoxification

## Abstract

Background: The medical literature reports health hazards for law enforcement personnel from repeated exposure to methamphetamine and related chemical compounds. Most effects appear transitory, but some Utah police officers with employment-related methamphetamine exposures developed chronic symptoms, some leading to disability. This report is of an uncontrolled retrospective medical chart evaluation of symptomatic officers treated with a sauna detoxification protocol designed to reduce the chronic symptoms and improve the quality of life. Methods: Sixty-nine officers consecutively entering the Utah Meth Cops Project were assessed before and after a treatment program involving gradual exercise, comprehensive nutritional support and physical sauna therapy. Evaluations included pre- and post-treatment scores of the Research and Development Corporation (RAND) 36-item Short Form Health Survey (SF-36) in comparison with RAND population norms, pre- and post-treatment symptom score intensities, neurotoxicity scores, Mini-Mental Status Examination, presenting symptom frequencies and a structured evaluation of treatment program safety. Results: Statistically significant health improvements were seen in the SF-36 evaluations, symptom scores and neurotoxicity scores. The detoxification protocol was well tolerated, with a 92.8% completion rate. Conclusions: This investigation strongly suggests that utilizing sauna and nutritional therapy may alleviate chronic symptoms appearing after chemical exposures associated with methamphetamine-related law enforcement activities. This report also has relevance to addressing the apparent ill effects of other complex chemical exposures. In view of the positive clinical outcomes in this group, broader investigation of this sauna-based treatment regimen appears warranted.

## Introduction

Persons addicted to methamphetamine develop serious health problems (Hall et al., 2003), but there is less understanding surrounding the high numbers of law enforcement personnel who experience significant symptoms associated with clandestine methamphetamine drug lab investigations (CDC, 2005). Responding to an active laboratory has been associated with a 7- to 15-time higher risk of becoming ill when compared to other activities with apparently lower chemical exposures (Burgess et al., 1996).

According to Marshall (2000), since 1993 ‘the number of clandestine drug laboratory investigations has continued to increase, making Utah the number one state for per capita methamphetamine laboratories.’ An estimated half of the approximately 300 Utah officers involved in methamphetamine lab-related activities later developed varying degrees of chronic illness and symptoms that were sometimes incapacitating (Personal communication from senior Utah officers). In 2005, local telecasts reported apparently higher rates of deaths, cancers, or unusual symptoms among these officers, implying a link between meth-lab-related exposures and subsequent health problems. While symptoms may be transitory, many individuals have persistent symptoms causing them to seek medical attention (Burgess, 2001; Burgess et al., 1996; Witter et al., 2007).

Methamphetamine synthesis utilizes hazardous chemicals including irritants and neurotoxins (Betsinger, 2006; Martyny et al., 2004). Aerosolized methamphetamine and associated toxic substances permeate carpets, walls and surfaces, and even wood construction (Martyny et al., 2005a,b). Disposed waste includes corrosive and other dangerous materials (EHP Forum, 1998). The capture, arrest, and transport of suspects and toxic materials can pollute officers’ clothing and vehicles (Martyny et al., 2005a,b).

These hazards were not well understood during the 1990’s upsurge of methamphetamine manufacture and lab raids. Officers often lacked personal protective equipment (PPE), including respiratory protection (CDC, 2000).

On-site detection of illicit substances included olfactory perception of drug-related odors. After securing the premises, officers commonly spent several hours on site, taking chemical inventories and samples for identification. Officers would often unknowingly pollute their cars while transporting chemicals and equipment to evidence storage sites or to the Drug Enforcement Administration (DEA) for analysis. Such contaminated vehicles could expose officers’ eyes, skin, and lungs for months afterward. Canine (K-9) handlers trained drug-sniffing dogs using residue-laden decoys, thus increasing the handler's methamphetamine exposures. Although preparation for chemical hazards has improved over time, toxic exposures can still occur.

The interactions of complex chemical mixtures may produce broad symptom patterns that developed in these officers: reduced respiratory function (Burgess et al., 2002); headache, cough, throat, and eye irritation; variable numbness; heartburn and esophageal reflux, skin rashes; and central nervous system symptoms (chronic headaches, irritability, severe insomnia, fatigue, and even psychosis) (Burgess, 2001; Burgess et al., 1996; CDC, 2000; Witter et al., 2007).

Precise concentrations of methamphetamine or the chemicals used for its production were not measured in this study, but these chemicals have serious health effects, even at low concentrations (Alexson and Hogstedt, 1994; Herpin et al., 2009). Use of recreational methamphetamine is clearly detrimental to human health (Hall et al., 2003; Schep et al., 2010). It appears plausible that exposure to methamphetamine (especially in illegal production laboratory settings) could possibly trigger subsequent chronic ill health but a causal link is not firmly established at present. Drug enforcement efforts obviously suffer when highly trained officers cannot function because of chronic disabling symptoms. However, whether a causal link between exposure and subsequent chronic illness becomes apparent or not, medical treatments to restore health and quality of life are clearly needed.

Appropriate treatment responses recognize and address chemical-related illnesses based on clinical signs or patterns (CDC, 2003). In 2007, the Utah Attorney General investigated a sauna-based detoxification regimen operating in Manhattan for treating chronically ill rescue and recovery workers exposed during the September 11, 2001 World Trade Center attack and collapse (Cecchini et al., 2006; Dahlgren et al., 2007). A senior police officer and a professional firefighter who were ill after methamphetamine lab-related exposures in Utah attributed substantial health improvement after receiving this treatment.

The treatment is a combination of modalities aimed at enhancing the broad elimination of body-stored toxic pollutants and improving symptoms common to chemical exposures, including those from illicit drug use (Hubbard, 2002). After the release of treatment protocol in 1979, Schnare et al. (1982) described the program’s safety and ability to reduce symptoms and improve mental functioning among individuals with a variety of chemical or illicit drug exposures.

A number of subsequent studies describe the safety of sauna-based regimens as well as their ability to reduce body stores of toxic pollutants and improve symptoms (Crinnion, 2007; Rea et al., 1996; Tsyb et al., 1998). Several studies using the Hubbard method showed significant reductions in human chemical pollutants and improvements in health (Schnare et al., 1984; Tretjak et al., 1989, 1990). Kilburn et al. (1989) found improvements in long-term memory, cognitive dysfunction, and peripheral neuropathy among the firefighters 6 months after PCB (polychlorinated biphenyl) exposures from burning transformers. The Hubbard detoxification procedure is used widely, including in substance abuse treatment, with evidence of its ability to eliminate chemical and drug residues (Cecchini and LoPresti, 2007).

The nonprofit American Detoxification Foundation (ADF) established and administered The Utah Meth Cops Project (UMCP), which uses the Hubbard detoxification protocol and monitors health and quality of life among Utah police officers, to address the symptoms consistent with (and appearing after) line-of-duty exposures to methamphetamine and related chemicals.

## Methods

### Description of the study group, inclusion, and exclusion criteria

This is a retrospective medical chart evaluation on the first 69 police officers sequentially entering the UMCP between October 2007 and July 2010. Officers were recruited through outreach efforts by project staff, word of mouth within the police community and referrals by their Chiefs of Police or County Sheriffs.

### Exclusion criteria

Pregnancy, known active cancer, being wheelchair-bound, a history of psychosis, extensive psychiatric treatment, or suicide attempts were the exclusion criteria.

### Inclusion criteria

(1) Law enforcement work within Utah, (2) documented contact with methamphetamine and related chemicals through law enforcement activities, and (3) the subsequent development of persistent medical symptoms or chronic ill health were the inclusion criteria. Officers gave written informed consent for treatment and outcomes monitoring, including reporting of aggregate findings.

The Medical Director included participants according to their comprehensive history and physical examination, EKG, and blood analysis (metabolic and liver panels, hepatitis B, C and HIV screen, complete blood count, and thyroid panel). Further tests were done, including testosterone levels, when direct questioning revealed problems that warranted evaluation. Officers with debilitating symptoms had some priority; no preferential treatment was given for the number of meth-related exposures, age, gender, or police rank.

Patients included undercover, narcotics, and Special Weapons and Tactics (SWAT) officers from many Utah urban and county jurisdictions, Utah Highway Patrol (UHP), Immigration and Customs Enforcement (ICE), officers affiliated with the DEA, and officers exposed while performing chemical laboratory analyses.

### Summary of the intervention

Treatment components include (1) 20–30 min of aerobic exercise, (2) comprehensive nutritional supplementation including increasing doses of niacin (vitamin B3), and (3) moderate-temperature sauna therapy, with breaks every 30 min for fluid and electrolyte restoration, totaling about 4 h daily. Treatment is given each day until maximum gains are achieved (typically 4–6 weeks). Daily progress is monitored by trained staff and recorded using a structured report form. The detailed protocol is described elsewhere (Hubbard, 2002; Schnare et al., 1982).

The 8-to-10 person sauna has ceramic tiles on all interior surfaces, with benches made of aged poplar wood. All surfaces were cleaned with a dilute fragrance-free soap solution, and eliminated through a floor S-drain. A tempered glass door provides direct assessment of sauna participants, and flow-through ventilation and dedicated ducting slowly evacuates sauna air outside the building.

The heat source (15,000 W capacity, 240 V, and 62.5 A) is a ceramic coil surrounded by granite stones, maintaining a stable 160°F temperature at 5 ft above the sauna floor. The heater emits a full spectrum of infrared wavelengths matching thousands of years of sauna use and protocol specifications (Hubbard, 2002).

### Outcome evaluations

Symptom changes and quality of life were assessed using a baseline history and physical examination, follow-up interviews, and a series of pre- and posttreatment assessments:

1. The RAND 36-item Short Form Health Survey (SF-36) assessed the 4-week health-related quality of life before treatment. The RAND SF-36 scoring mechanism differs from that licensed by Medical Outcomes Trust and produces a 9-scale profile of functional ability and physical and mental well-being. SF-36 scores were also compared pre- and post-treatment and to RAND US adult population norms (Hays et al., 1993).
2. A 50-item pre- and post-treatment survey of the preceding 4 weeks’ symptoms, sick days, and sleep patterns was developed by the Foundation for Advancements in Science and Education (FASE) for clinical settings using the Hubbard regimen.
3. A 13-item pre- and post-treatment neurotoxicity questionnaire based on the parameters of Singer (2006) rated the preceding 3 weeks’ problems involving irritability, social withdrawal, decreased motivation, recent memory, concentration, mental slowness/fog, sleep disturbances, fatigue, frequency and severity of headaches, sexual dysfunction, extremity numbness, and decreased mental sharpness, expressed on a 0–10 Likert-type scale.
4. The Mini-Mental State Examination (Folstein et al., 1975).
5. Daily report form: a structured summary of vital signs/events recorded by trained staff on each treatment day, including any undesirable effects (whether or not related to treatment). For safety evaluation, any adverse events or interruptions of the protocol appear on the daily report form and are assessed by the medical director.

Analyses compared the means and standard deviations of test scores before and after program participation. Statistical significance was calculated using 2-tailed Student *t* test with paired scores for pre/post comparisons or unpaired for comparison with RAND SF-36 published norms. Two nonparametric measures of statistical significance were also calculated for paired data sets: Wilcoxon matched pairs test and the sign test.

## Results

### Treatment length and completion rates

A total of 66 men and 3 women (*n* = 69), averaging 44.6 years of age (range: 31–60 years) enrolled sequentially with a 92.8% completion rate; 5 men did not complete the treatment. Mean treatment length for the 64 patients who completed the treatment was 33 days (range: 15–56 days); for the 5 who did not complete the treatment, the mean treatment length was 29 days (range: 23–25 days). Reasons for discontinuation are summarized in Table 2.

**Table 1.** Meth Cops mean RAND SF-36 category scores and standard deviation before and after treatment (*p* < 0.001 for all categories) and compared with RAND published norms^a^

	Before treatment, *n* = 61		After treatment, *n* = 61		RAND norm *n* = 2471		Significance to RAND norm (*p* value)	
	Mean	SD	Mean	SD	Mean	SD	Before^b^	After^c^
Physical functioning	79.01	17.05	92.78	10.97	70.61	27.42	0.017	<0.001
Role limitations due to								
Physical health	53.28	38.86	85.25	29.71	52.97	40.78	0.953	<0.001
Emotional problems	56.28	38. 75	90.16	23.84	65.78	40.71	0.072	<0.001
Energy/fatigue	35.90	19.74	68.61	18.31	52.15	22.39	<0.001	<0.001
Emotional well-being	62.16	18.00	85.05	10.30	70.38	21.97	0.004	<0.001
Social functioning	65.37	22.29	85.86	20.35	78.77	25.43	<0.001	0.031
Pain	59.39	19.55	82.17	18.46	70.77	25.48	<0.001	<0.001
General health	49.41	18.56	78.44	13.68	56.99	21.11	0.006	<0.001
Year ago health change	45.49	19.64	85.25	19.57	59.14	23.12	<0.001	<0.001

^a^Statistical significance was accepted at *p *< 0.05. All SF-36 mean pre-detoxification health scores were statistically lower (*p* < 0.001) compared with mean post-treatment scores, based on paired two-tailed *t* test.

^b^Mean SF-36 pre-detoxification health scores were statistically less than the published population norms using RAND methodology, except *role limitations due to physical health* (*p *= 0.953) and *role limitations due to emotional problems* (*p* = 0.072), based on the two-tailed *t* test.

^c^Mean SF-36 post-treatment health scores were all significantly higher than the published RAND methodology population norms.

### Symptoms present in more than 50% of the officers (n = 66)

**Figure 1.**
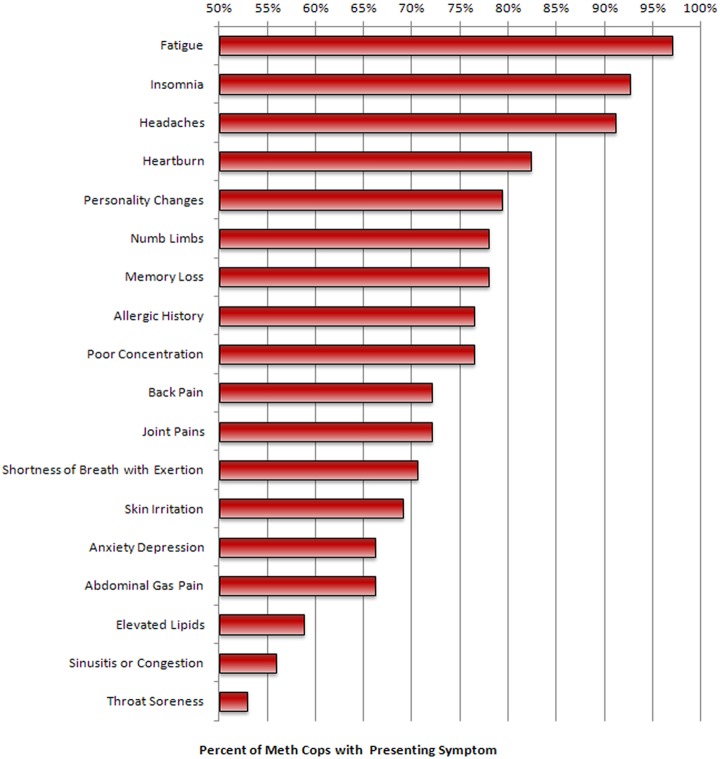
Percentage of Utah Meth Cops Study group reporting specific symptoms (symptoms displayed were reported by >50% of the officers.) *n* = 66. See narrative text for frequency data of additional symptoms.

The enrollment evaluation is shown in Figure 1, which includes fatigue: 96%, insomnia: 91%, headaches: 90%, heartburn: 81%, personality changes: 78%, numbness in hands and/or feet: 77%, memory loss: 77%, allergic history: 75%, poor concentration: 75%, back pain: 71%, joint pains: 71%, shortness of breath on exertion: 70%, skin irritation: 68%, anxiety/depression, 65%; abdominal gas/pain: 65%, sinusitis/congestion: 55%, and sore throat: 52%.

### Symptoms present in less than 50% of the officers (n = 66)

The symptoms include hearing loss: 46%, lower libido: 43%, chronic cough: 41%, slower healing: 41%, cardiac palpitations: 39%, history of prior loss of consciousness: 39%, eye irritation: 39%, dizziness: 39%, diarrhea: 36%, history of medication allergy or intolerance: 33%, sudden hair loss: 22%, smell loss: 19%, and ear ringing: 4%.

### Percentage of officers with abnormal findings

The abnormal findings include elevated blood lipids: 58%, elevated liver function tests: 41%, positive rombergism (inability to maintain balance in a ‘tandem stance’ without visual input): 35%, hypertension: 28%, high blood glucose: 19%, low blood testosterone: 17%, and low blood thyroid: 17%.

### RAND SF-36 scores: change in health-related quality of life (n = 61)

**Figure 2.**
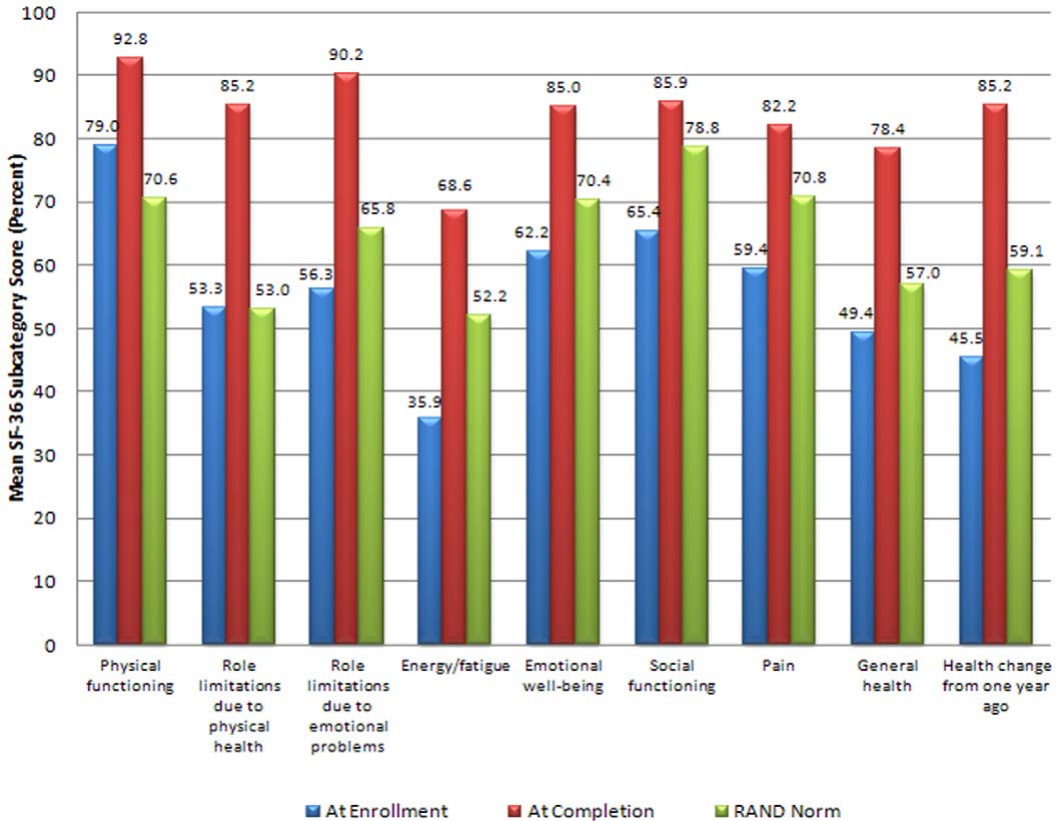
Meth Cops RAND 36-item Short Form Health Survey (SF-36) health status pre- and postdetoxification therapy, in comparison with RAND population norms. *n* = 61. Means at treatment enrollment compared with completion produced significance at *p *< 0.001 for all subscales, using paired two-tailed Student *t* test.

Table 1 displays the mean pre- and post-treatment SF-36 scores calculated using RAND methodology and compared with US population norms for those officers who completed the regimen. Figure 2 shows the data in graphic form.

**Table 2.** Regimen safety: adverse events experienced during the sauna protocol

	*n* = 69 patients (patients may experience multiple events)			
	Number who experienced event	Number who missed days due to event	Number requesting medical consult due to event	Number who discontinued program due to event
Niacin flush, itchy skin	69	0	0	0
Emotional, irritable, despondent	18	0	0	0
Cough, congestion, sore throat	13	0	0	0
Flu-like symptoms, no fever	11	0	0	0
Flu-like symptoms with mild fever	2	0	0	0
Headache	6	0	0	0
Sleeplessness, vivid dreams	15	12^a^	0	1^b^
Fatigue	14	0	0	0
Stomach cramps, nausea, diarrhea	8	3	0	0
Body aches	5	2	0	0
Gout	2^c^	2	1	1
Work or other schedule conflicts	5	4	0	3^d^

^a^Per protocol, patients who achieve less than 6.5 h of sleep have their next day’s treatment shortened to 10 min of exercise and 4 sauna sessions of 10 min each separated by 10-min breaks.

^b^This patient reported substantial health improvement but had insufficient sleep throughout the program. Treatment is considered incomplete for purposes of all data analyses.

^c^Both patients reported episodes of gout prior to starting the regimen.

^d^Two officers allotted insufficient treatment time and had to return to work; the third discontinued, citing work-related factors and also missed 6 days in the middle of the regimen.

Mean values of the officer pre-treatment health-related quality of life scores were significantly lower than RAND population norms (*p* < 0.001) in all nine subscales except *role limitations due to physical health* (*p* = 0.953) and *role limitations due to emotional problems* (*p* = 0.072). Post-treatment, the officers’ scores showed statistically significant improvements when compared with pre-treatment scores (*p* < 0.001 across all subscales, by either parametric *t* test or nonparametric comparisons). Officers’ post-treatment scores were also significantly improved for all subscales compared with RAND population norms (*p* < 0.001 for all subscales, except *social functioning* at *p* = 0.031).

### Symptom severity and poor health days (n = 62)

**Figure 3.**
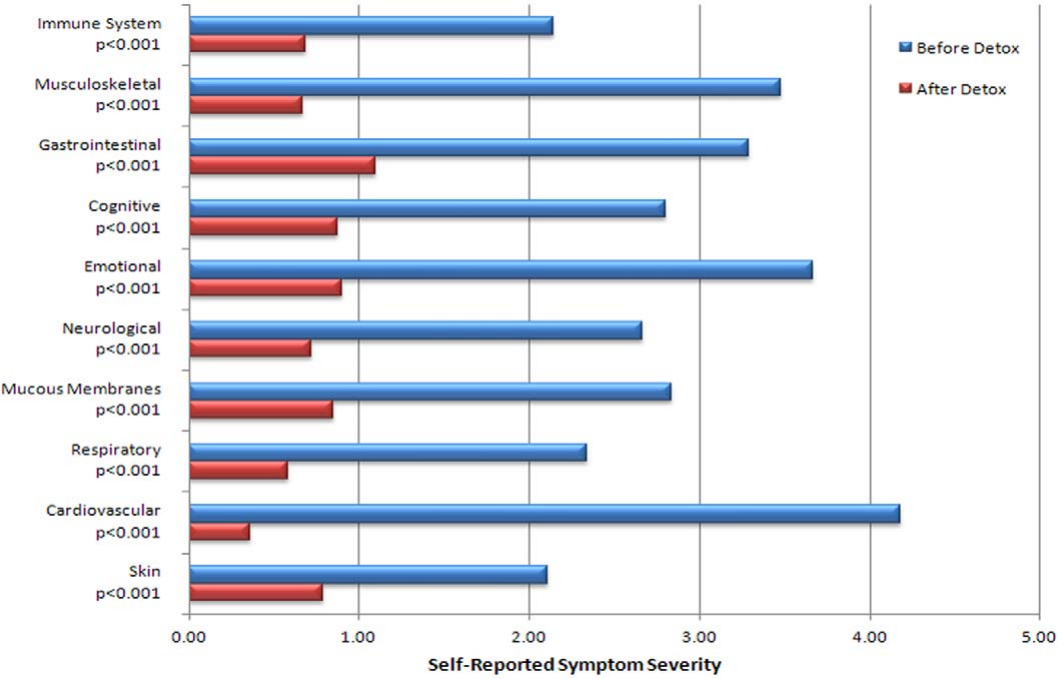
Symptom severity pre- and postdetoxification therapy. Reduction in symptom severity with detoxification, *n* = 67. *p* values based on two tailed *t *test of paired samples. Comparison of Meth Cops symptom severity before and after leaving treatment. Mean scores was significant at *p *< 0.001 for all scales using paired two-tailed Student *t* test. Note: these data include 3 participants who had not fully completed the regimen.

Mean pre- and post-treatment symptom severity scores are shown in Figure 3 and are significantly reduced post-treatment versus pre-treatment (*p* < 0.001 for all subscales).

Patients reported the number of days of ‘poor health,’ ‘limited activities,’ and ‘sick days’ in the 30-day pre-treatment period versus the 30-day period before finishing (i.e. the treatment period). Patients reported means of (A) 9.3 days of *poor physical health *pre-treatment, improving to 1.8 days by completion; (B) 6.3 days of *poor mental health *pre-treatment versus 1.4 days by completion; (C) 4.3 days of *limited activities due to poor health* pre-treatment versus 0.2 days by completion; and (D) 2.0 *sick days* pretreatment versus 0.3 days by completion (data table not displayed, *n* = 62, *p* < 0.001 for each comparison).

### Sleep patterns

Participants averaged 5.8 h of sleep per night pre-treatment, which improved to 7.6 h on completion (data table not displayed, *n* = 62, *p* < 0.001).

### Neurotoxicity scores (n = 38)

This questionnaire was administered from officer #20 onward. Excluding incomplete data, there were 38 matched pairs of pre- and post-treatment responses (84.4% response rate). The mean pre-treatment neurotoxicity score was 65.5 (SD = 24.8), while the post-treatment mean score was 14.6 (SD = 11.5, *p* < 0.001).

### Mini-Mental Status evaluation (n = 38)

On a 30-point scale, scores below 25 indicate significant cognitive dysfunction. No measurable change was detected comparing mean pre- and post-treatment scores (29.3 vs. 29.1, respectively; *p* = 0.122).

## Discussion

Police officers generally require robust physical qualifications and emotional stability. In contrast to job selection criteria, the officers treated in this project had chronic debilitating symptoms consistent with chemical exposures (Carpenter et al., 2002).

Of concern in clandestine meth labs are chemicals including phosphine, iodine, hydrogen chloride and solvents (acetone, toluene, charcoal lighter fluid, and Coleman fuel), as well as methamphetamine and its precursors (Martyny et al., 2004). Each has the potential for adverse symptoms, even at low exposure levels.

Persons exposed to illegal methamphetamine production commonly experience headaches, respiratory and eye irritation, nausea, and vomiting (Thrasher et al., 2009). Prolonged methamphetamine exposure is also associated with neurotoxic effects, including neuropathy, amyotrophic lateral sclerosis (ALS) and Parkinson’s disease (Garwood et al., 2006), suggesting possible neurotoxic effects.

In addition to immediate symptoms, knowledge of toxicity pathways and the downstream effects of complex chemical exposures are increasing. Many of the precursor solvents as well as ephedrine, pseudoephedrine, and methamphetamine, have lipophilic properties consistent with bioaccumulation, contributing to their potential for long-term adverse health effects. Levisky et al. (2001) showed the deposition of methamphetamine and related chemicals in human tissues.

Many environmental chemicals can disrupt thyroid hormone levels or have antiandrogenic effects (Woodruff, 2010). Estrogenic pollutants alter androgen levels and might be implicated in these officers’ symptoms and laboratory findings (Sharpe, 2003; Wu et al., 2010).

In this small group of 69 individuals, it is surprising that 2 subsets of 17% of patients showed low thyroid and/or low testosterone status. Prevalence of hypothyroidism in the United States is about 5% (Hollowell et al., 2002). A preexisting thyroid imbalance may predispose officers to chronic illness, but low thyroid status may have directly resulted from methamphetamine-related exposures, in light of the causal relationship between environmental chemicals and low thyroid function (Miller et al., 2009).

Also unusual was the *symptoms in common* among those reporting chronic ill health. More than 75% of officers reported all the following nine symptoms: fatigue, insomnia, headaches, heartburn, personality changes, numbness in hands and/or feet, memory loss, a prior history of allergy symptoms, and poor concentration. This symptom cluster raises the possibility that ‘exposures in common’ may have triggered ‘symptoms in common.’ This symptom pattern may help future researchers or treatment professionals better recognize or classify methamphetamine-related exposures. The ‘pre-treatment’ SF-36 scores of methamphetamine-exposed officers indicated more pain, more fatigue, and significantly poorer health than the general population.

The ‘Hawthorne effect’ (improvements arising solely from the ‘care and attention’ given and not the treatment itself) (Leonard 2008) should be considered given that many of these officers were told their health was ‘normal’ and/or the symptoms ‘were all in their head.’ However, this effect is unlikely to be playing any significant role given the prolonged nature and magnitude of objective improvements and also that the SF-36 health scores and symptom severity profiles were not only statistically improved, there was consistent improvement across all sub-scales.

When evaluated individually, some officers might appear to have a psychiatric diagnosis. When viewed collectively, however, the pattern could just as reasonably be interpreted as neurotoxic in origin. The Centers for Disease Control (2003) states. ‘Treating exposed persons by chemical syndrome rather than by specific agent probably is the most pragmatic approach to the treatment of illnesses caused by chemical exposure.’

It is within this context that the Hubbard sauna-based treatment protocol was utilized. If chemical exposures and/or contamination caused these chronic symptoms, then a multifaceted ‘detoxification’ program was a reasonable approach.

To our knowledge, this is the first time a sauna-based ‘detoxification program’ has been evaluated in methamphetamine-exposed police officers. The vast majority completed the regimen with minimal discomfort or inconvenience, achieving significant reductions in their symptoms and measurably improved the health and quality of life. This suggests that this program could help similarly exposed police officers elsewhere.

Because law enforcement personnel will likely continue to have exposures to methamphetamine and associated chemicals, these study data suggest that thyroid and androgen dysfunction and neurotoxic effects might appropriately be assessed. Those with chronic symptoms may benefit from the treatment programs (such as this one) that have been shown to improve health and reduce residual pollutant chemical burden. Despite the obvious limitations of this preliminary study, including the lack of matched controls, the clinical outcomes make a case for continued investigation of the sauna-based Hubbard detoxification protocol.

## References

[bibr1-0748233711425070] Alexson O Hogstedt C (1994) The health effects of solvents. In: Zenz C Dickerson OB Horvath EP (eds) Occupational Medicine. St. Louis: Mosby Press, 764–768

[bibr2-0748233711425070] Betsinger G (2006) Coping with meth lab hazards. Occupational Health and Safety 75(11): 50, 52, 54–58 17125087

[bibr3-0748233711425070] Burgess JL (2001) Phosphine exposure from a methamphetamine laboratory investigation. Journal of Toxicology. Clinical Toxicology 39(2): 165–168 11407503 10.1081/clt-100103833

[bibr4-0748233711425070] Burgess JL Barnhart S Checkoway H (1996) Investigating clandestine drug laboratories: adverse medical effects in law enforcement personnel. American Journal of Industrial Medicine 30(4): 488–494 8892555 10.1002/(SICI)1097-0274(199610)30:4<488::AID-AJIM15>3.0.CO;2-0

[bibr5-0748233711425070] Burgess JL Kovalchick DF Siegel EM Hysong TA McCurdy SA (2002) Medical surveillance of clandestine drug laboratory investigators. Journal of Occupational and Environmental Medicine 44(2): 184–189 11851220 10.1097/00043764-200202000-00014

[bibr6-0748233711425070] Carpenter DO Arcaro K Spink DC (2002) Understanding the human health effects of chemical mixtures. Environmental Health Perspective 110(suppl 1): 25–42 10.1289/ehp.02110s125 PMC1241145 11834461

[bibr7-0748233711425070] CDC (2000) Public health consequences among first responders to emergency events associated with illicit methamphetamine laboratories—selected states, 1996-1999. MMWR Morbidity and Mortality Weekly Report 49(45): 1021–1024 11098778

[bibr8-0748233711425070] CDC (2003) Recognition of illness associated with exposure to chemical agents—United States, 2003. MMWR Morbidity and Mortality Weekly Report 52(39): 938–940 14523372

[bibr9-0748233711425070] CDC (2005) Acute public health consequences of methamphetamine laboratories—16 states, January 2000-June 2004. MMWR Morbidity and Mortality Weekly Report 54(14): 356–359 15829865

[bibr10-0748233711425070] Cecchini M LoPresti V (2007) Drug residues store in the body following cessation of use: impacts on neuroendocrine balance and behavior—use of the Hubbard sauna regimen to remove toxins and restore health. Medical Hypotheses 68(4): 868–879 17045758 10.1016/j.mehy.2006.08.035

[bibr11-0748233711425070] Cecchini MA Root DE Rachunow JR Gelb PM (2006) Chemical exposures at the World Trade Center: use of the Hubbard sauna detoxification regimen to remove toxins and restore health. Townsend Letter 273: 58–65

[bibr12-0748233711425070] Crinnion W (2007) Components of practical clinical detox programs—sauna as a therapeutic tool. Alternative Therapies in Health and Medicine 13(2): S154–S156 17405694

[bibr13-0748233711425070] Dahlgren J Cecchini M Takhar H Paepke O (2007) Persistent organic pollutants in 9/11 world trade center rescue workers: reduction following detoxification. Chemosphere 69(8): 1320–1325 17234251 10.1016/j.chemosphere.2006.05.127

[bibr14-0748233711425070] EHP Forum (1998) The threat of meth. Environmental Health Perspectives 106: A172–A173

[bibr16-0748233711425070] Folstein MF Folstein SE McHugh PR (1975) “Mini-mental state”. A practical method for grading the cognitive state of patients for the clinician. Journal of Psychiatric Research 12(3): 189–198 1202204 10.1016/0022-3956(75)90026-6

[bibr15-0748233711425070] Garwood ER Bekele W McCulloch CE Christine CW (2006) Amphetamine exposure is elevated in Parkinson’s disease. Neurotoxicology 27(6): 1003–1006 16620991 10.1016/j.neuro.2006.03.015

[bibr17-0748233711425070] Hall HV McPherson SB Twemlow SW Yudko E (2003) Epidemiology. In: Yudko E Hall HV McPherson SB (eds) Methamphetamine Use: Clinical and Forensic Aspects. Boca Raton: CRC Press, 13–15

[bibr18-0748233711425070] Hays RD Sherbourne CD Mazel RM (1993) The RAND 36-Item Health Survey 1.0. Health Economics 2(3): 217–227 8275167 10.1002/hec.4730020305

[bibr19-0748233711425070] Herpin G Gargouri I Gauchard GC Nisse C Khadhraoui M, Elleuch B, et al. (2009) Effect of chronic and subchronic organic solvents exposure on balance control of workers in plant manufacturing adhesive materials. Neurotoxicity Research 15(2): 179–186 19384580 10.1007/s12640-009-9018-0

[bibr20-0748233711425070] Hollowell JG Staehling NW Flanders WD Hannon WH Gunter EW, Spencer CA, et al. (2002) Serum TSH, T(4), and thyroid antibodies in the United States population (1988 to 1994): National Health and Nutrition Examination Survey (NHANES III). The Journal of Clinical Endocrinology and Metabolism 87(2): 489–499 11836274 10.1210/jcem.87.2.8182

[bibr21-0748233711425070] Hubbard LR (1990) Clear Body Clear Mind. 2002 ed Los Angeles: Bridge Publications

[bibr22-0748233711425070] Kilburn KH Warsaw RH Shields MG (1989) Neurobehavioral dysfunction in firemen exposed to polycholorinated biphenyls (PCBs): possible improvement after detoxification. Archives of Environmental Health 44(6): 345–350 2514627 10.1080/00039896.1989.9935904

[bibr27-0748233711425070] Leonard KL 2008 Is patient satisfaction sensitive to changes in the quality of care? An exploitation of the Hawthorne effect. Journal of Health Economics 27(2): 444–59 18192043 10.1016/j.jhealeco.2007.07.004

[bibr23-0748233711425070] Levisky JA Bowerman DL Jenkins WW Johnson DG Karch SB (2001) Drugs in postmortem adipose tissues: evidence of antemortem deposition. Forensic Science International 121(3): 157–160 11566418 10.1016/s0379-0738(01)00397-8

[bibr24-0748233711425070] Marshall DR (2000) Report before the 106th congress: emerging drug threats and perils facing Utah’s youth. Salt Lake City, UT: Committee on the Judiciary, United States Senate http://frwebgate.access.gpo.gov/cgi-bin/getdoc.cgi?dbname=106_senate_hearings&docid=f:73821.pdf (accessed 17 April 2011)

[bibr25-0748233711425070] Martyny JW Arbuckle SL McCammon CS Esswein EJ Erb N (2004) Chemical exposures associated with clandestine methamphetamine laboratories . Denver, CO : National Jewish Medical and Research Center www.nationaljewish.org/pdf/chemical_exposures.pdf (accessed 17 April 2011).

[bibr26-0748233711425070] Martyny JW Van Dyke MV McCammon CS Erb N Arbuckle SL (2005a) Chemical exposures associated with clandestine methamphetamine laboratories using the anhydrous ammonia method of production. Denver, CO: National Jewish Medical and Research Center http://www.njc.org/pdf/Ammonia%20Meth.pdf . (accessed 17 April 2011)

[bibr41-0748233711425070] Martyny JW Van Dyke M McCammon CS Erb N Arbuckle SL (2005b). Chemical exposures associated with clandestine methamphetamine laboratories using the hypophosphorous and phosphorous flake method of production. National Jewish Medical Research Center http://www.njc.org/pdf/meth-hypo-cook.pdf (Accessed 9 Feb 2011)

[bibr42-0748233711425070] Miller MD Crofton KM Rice DC Zoeller RT (2009) Thyroid-disrupting chemicals: interpreting upstream biomarkers of adverse outcomes. Environmental Health Perspectives 117(7): 1033–1041 19654909 10.1289/ehp.0800247 PMC2717126

[bibr28-0748233711425070] Rea WJ Pan Y Johnson AR Ross GH Suyama H Fenyves EJ (1996). Reduction of chemical sensitivity by means of heat depuration, physical therapy and nutritional supplementation. Journal of Nutritional and Environmental Medicine 6: 141–148

[bibr29-0748233711425070] Schep LJ Slaughter RJ Beasley DM (2010) The clinical toxicology of metamfetamine. Clinical Toxicology (Philadelphia) 48(7): 675–694 10.3109/15563650.2010.516752 20849327

[bibr30-0748233711425070] Schnare DW Ben M Shields MG (1984) Body burden reduction of PCBs, PBBs and chlorinated pesticides in human subjects. Ambio 13: 378–380

[bibr31-0748233711425070] Schnare DW Denk G Shields M Brunton S (1982) Evaluation of a detoxification regimen for fat stored xenobiotics. Medical Hypotheses 9(3): 265–282 7144634 10.1016/0306-9877(82)90156-6

[bibr32-0748233711425070] Sharpe RM (2003) The ‘oestrogen hypothesis’- where do we stand now? International Journal of Andrology 26(1): 2–15 12534932 10.1046/j.1365-2605.2003.00367.x

[bibr33-0748233711425070] Singer R (2006) Neurotoxicity Guidebook. San Diego, CA: Aventine Press, 3

[bibr34-0748233711425070] Thrasher DL Von Derau K Burgess J (2009) Health effects from reported exposure to methamphetamine labs: a poison center-based study. Journal of Medical Toxicology 5(4): 200–204 19876853 10.1007/BF03178267 PMC3550415

[bibr35-0748233711425070] Tretjak Z Beckmann S Tretjak A Gunnerson C (1989) Report on occupational, environmental, and public health in Semic: a case study of polychlorinated biphenyl (PCB) pollution. In: Post-Audits of Environmental Programs and Projects; Proceedings, Environmental Impact Analysis Research Council / ASCE. New Orleans, LA, 57–72

[bibr36-0748233711425070] Tretjak Z Shields M Beckmann SL (1990) PCB reduction and clinical improvement by detoxification: an unexploited approach? Human and Experimental Toxicology 9(4): 235–244 2143911 10.1177/096032719000900406

[bibr37-0748233711425070] Tsyb AF Parshkov EM Barnes J Yarzutkin VV Vorontsov NV Dedov VI (1998) Proceedings of the 1998 International Radiological Postemergency Response Issues Conference. Washington, DC: US EPA, 162–166, efile pages 178–182

[bibr38-0748233711425070] Witter RZ Martyny JW Mueller K Gottschall B Newman LS (2007) Symptoms experienced by law enforcement personnel during methamphetamine lab investigations. Journal of Occupational and Environmental Hygiene 4(12): 895–902 17943587 10.1080/15459620701693516

[bibr39-0748233711425070] Woodruff TJ (2011) Bridging epidemiology and model organisms to increase understanding of endocrine disrupting chemicals and human health effects. The Journal of Steroid Biochemistry and Molecular Biology 127(1–2): 108–117 21112393 10.1016/j.jsbmb.2010.11.007 PMC6628916

[bibr40-0748233711425070] Wu FC Tajar A Beynon JM Pye SR Silman AJ Finn JD (2010) Identification of late-onset hypogonadism in middle-aged and elderly men. The New England Journal of Medicine 363(2): 123–135 20554979 10.1056/NEJMoa0911101

